# Child health, nutrition and gut microbiota development during the first two years of life; study protocol of a prospective cohort study from the Khyber Pakhtunkhwa, Pakistan

**DOI:** 10.12688/f1000research.158415.1

**Published:** 2024-11-07

**Authors:** Muhamamd Shahzad, Muhammad Ismail, Benjamin Misselwitz, Ahsan Saidal, Simon C Andrews, Khalid Iqbal, Hatice Akarsu, Ziad Al Nabhani

**Affiliations:** 1 Institute of Basic Medical Sciences, Khyber Medical University, Peshawar, Pakistan; 2 Faculty of Dentistry, Zarqa University, Zarqa, Jordan; 3 Maurice Müller Laboratories, Department for Biomedical Research, University of Bern, Bern, Switzerland; 4 Department of Visceral Surgery and Medicine, Bern University Hospital, Bern, Switzerland; 5 Institute of Health Sciences, Khyber Medical University, Swat, Pakistan; 6 School of Biological Sciences, University of Reading, Reading, UK; 7 Epidemiological Methods and Etiological Research, Leibniz Institute for Prevention Research and Epidemiology, Bremen, Germany; 8 SIB Swiss Institute of Bioinformatics, Lausanne, Switzerland

**Keywords:** Microbiome, Infants, Growth, Vulnerable, Malnutrition

## Abstract

Recent evidence suggests that the development of gut microbiota during infancy affects several metabolic, immune, and endocrine pathways in humans. An imbalance in gut microbiota diversity or function, also known as dysbiosis, not only affects early child growth and development, but is also linked with the development of chronic, non-communicable diseases in later life. The **C**hild **H**ealth **A**nd **M**icrobiome Development Study – **P**akistan (CHAMP) study aimed to longitudinally assess gut microbiota development and associated factors (maternal, child, and demographic) during early childhood in populations residing in malnutrition-endemic communities in Pakistan. A prospective cohort of mother-infant pairs (n=70) will be recruited from District Swat, Pakistan, and followed for two years. Complete information about demographic characteristics, anti-natal and post-natal care, dietary intake, feeding practices, and child health will be collected at baseline and 3, 6, 12, 18, and 24 months. Anthropometric measurements (height, weight, mid-upper arm circumference, and head circumference), dry blood spots, and fecal samples were also collected. Ethical approval for the study was obtained from Khyber Medical University, Pakistan. The study is also registered on clincaltrial.gov (Ref no: NCT05793294). The study findings will help researchers understand gut microbiota development, associated factors, and their impact on longitudinal growth in infants during the first two years of life.

## Introduction

Infancy, the period from birth to around two years of age, is a crucial stage in human development, characterized primarily by rapid physical, mental, and emotional growth. ^1^ During this period, many environmental factors such as gestational age, sex, ethnicity, dietary intake, nutritional status, and socioeconomic conditions significantly affect child growth, development, and overall health. ^2, 3^ However, to date, these factors, either alone or synergistically, have failed to explain the overall spectrum and variations in children. Recent studies have emphasized that apart from the intersection of environmental factors, the gut microbiome also plays an important role in mediating growth and development, especially during infancy.

The human fetus is believed to develop in a microbe-free environment. ^4^ However, immediately after birth, the infant gut, especially the distal ileum and colon, is rapidly colonized by different microorganisms (bacteria, fungi, archaea, and viruses) via vertical transmission from maternal body sites such as the vagina, skin, oral cavity, and gut. ^5^ In newborn infants, the gut microbiota exhibits low diversity, consisting mainly of facultative anaerobic bacteria, such as *Proteobacteria* (e.g., *Escherichia* and *Enterobacter*) and *Firmicutes* ( *Staphylococcus*, *Enterococcus*and, and *Streptococcus*). ^6^ Early colonization has a profound influence on the gut environment and subsequent colonization by obligate anaerobes, such as *Bifidobacterium, Bacteroides* and *Clostridium.* The relative abundance of *Bifidobacteria* gradually increases 3 – 4 days after birth and becomes a predominant bacterial genus when the baby is approximately 1 month old. ^7, 8^ In healthy growing children, the gut microbiota gradually increases in complexity with a highly diverse microbial composition during and after the first two–three years of life. ^9^ During this period, the patterned ecological assembly of microbes occurs mainly under the influence of delivery mode, type of feeding, complimentary feeding practices, antibiotic use, and geography. ^9– 12^ Gut microbiota development during early life affects several metabolic, immune, and endocrine pathways and is thus intimately linked to child growth and development. ^13^ Therefore, any disturbances in the host-microbe coevolution, microbial composition, or functional potential of the gut microbiome, commonly referred to as microbial dysbiosis, can impair immune function, growth, and development in children.

In recent years, several studies have reported associations between gut microbiome dysbiosis and impaired growth and development in children from different countries across the word. For example, a causal relationship between gut microbiome immaturity, undernutrition, and impaired growth has been reported in 6- and 18-months old children. ^14^ Similarly, a Swedish cohort encompassing 471 children has also reported significantly lower microbial diversity and reduced abundance of *Faecalibacterium* and *Ruminococcus* in the gut microbiome of children with slower weight gain compared to normal children. ^15^ Research studies from developing countries, where malnutrition is common, have also reported impaired or immature gut microbiome in malnourished (stunting, wasting, and underweight) children compared to that in healthy controls. In Bangladesh, children with severe acute malnutrition (SAM) exhibit a considerably less diverse gut microbiome, low relative abundance of *Bacteroidetes* and high relative abundance of *Proteobacteria* especially the pathogen/pathobiont genera such as *Klebsiella, Escherichia, Shigella*, and *Streptococcus*. ^16^ In another study of a group of SAM infants aged 6–24 months, the absolute abundance of *B. infantis* was considerably lower than that of age-matched healthy infants. ^17^ These findings were also confirmed by studies conducted in other developing countries in Asia and Africa. ^18– 20^ Altogether, these findings suggest that an immature/poorly developed gut microbiome in infants and young children is associated with malnutrition and its consequences, particularly impaired growth and development. As a result, a nutritional intervention strategy that does not consider the gut microbiome will fail to ameliorate the long-term consequences of malnutrition, including child health and cognitive development. ^21^ Clinical studies in animals and humans have already demonstrated the positive impact of microbiome-based nutritional interventions on the gut microbiome and ponderal growth. ^22, 23^ These findings may have important implications for low- and middle-income countries such as Pakistan, where malnutrition in children is a crucial public health challenge.

Pakistan is the 5 ^th^ most populous country in the world (approximately 250 million people) ^24^ with enormous economic, geopolitical, social, and poverty-related challenges. ^25^ According to the latest national nutrition survey, the prevalence of stunting (40.2%), wasting (17.7%), and micronutrient deficiencies in children under five years of age ^26^ remained unacceptably high. Over the last five years, the country has been placed in the “Serious” category of the Global Hunger Index. ^27^ The consequences of malnutrition are also economically catastrophic with $7.6 billion/annum (~3% of GDP) lost to malnutrition. ^28^ Over the last two decades, there has been virtually no improvement in the overall nutritional status of the Pakistani population, despite efforts from the government and developmental organizations. Without coordinated efforts, it is very unlikely that Pakistan will achieve the UN Sustainable Development Goals to end hunger and all forms of malnutrition by 2030. ^29^ Successful tackling this issue requires cost-effective, culturally relevant, and sustainable nutritional interventions. Keeping in mind the central role of the gut microbiome in mediating malnutrition in children, it is crucial to map the compositional and functional dynamics of the gut microbiome and associated maternal, child, and environment-related factors during early childhood in populations residing in malnourished communities in Pakistan. Therefore, the current study attempts to characterize how the gut microbiome develops in a cohort of children at high risk of malnutrition in the Khyber Pakhtunkhwa province of Pakistan.

## Protocol

### Objectives

The primary objective of the CHAMP study is to characterize gut microbiota development and longitudinal growth in infants during the first two years of life. This will be done by assessing the microbial diversity, relative abundance of the dominant microbiota, and interindividual variations in microbial diversity and infant growth patterns. The secondary objective of the study is to investigate the impact of (i) sociodemographic characteristics, (ii) mode of delivery, (iii) maternal factors such as diet and anti-natal and post-natal care, (iv) child dietary intake and feeding practices, and (v) nutritional status of the child on gut microbiota composition at different time points from birth until the child is two years old.

### Operational definitions

• **Nutritional status:** The nutritional status of the child will be measured in terms of weight for age (WAZ), height for age (HAZ), and weight for height (WHZ) according to the WHO reference range. ^30^
• **Underweight:** Defined as weight for age of the child below two standard deviations (-2SD) from the median weight for age of the reference population.
• **Stunting:** Child height for age below minus two standard deviations (-2SD) from the median height for the age of the reference population.
• **Wasting:** Child weight for height below minus two standard deviations (-2SD) from the median weight for height of the reference population.

### Study design and setting

The CHAMP study was a population-based, prospective cohort study involving mother-infant pairs (dyads) from District Swat, Pakistan. Swat is located at 34°46′58″ N and 72°21′43″ N in Khyber Pakhtunkhwa province of Pakistan and is home to more than 2.3 million people. ^31^ Because of its geographic location in the Hindukush–Himalayan region, most of the land is occupied by mountains and thick forests. The area is one of the most marginalized and neglected regions of the province, with livestock, agriculture, horticulture, and tourism as the primary sources of income and livelihood of the local population. ^32^ Swat is among the most vulnerable districts in Pakistan and is prone to climate change. Since 2010, the district has been hit by at least three major floods that have significantly disrupted essential services, such as healthcare, sanitation, and access to safe drinking water. ^33^ As a result, the majority of the population, especially children, are at a high risk of malnutrition and its deleterious consequences.

Administratively, the district is divided into seven administrative units known as Tehsil Municipal Administration units (TMAs). Tehsil Matta was chosen as the study area because it is the second most populated tehsil in Swat district, with the highest number of rural and remote communities. Following a preliminary survey, UC Beha was selected as the study site for several reasons. It is located at a high altitude with the longest adjoining borders with district Dir (Upper), and most of the population lives in agropastoral communities with very limited access to education and healthcare facilities. In winter, access to the area is limited by heavy snowfall and landslides.

### Sampling technique

District swats are divided into seven administrative units known as Tehsil Municipal Administration units (TMAs). Of these, tehsil Matta was chosen as the study area because it is the second most populated tehsil in Swat district, located at a high altitude with the longest adjoining borders with Dir district (Upper), which has the highest number of rural and remote communities. The Matta tehsil is further divided into union ouncils, which are the primary administrative institutions in Pakistan. In rural areas, union councils are referred to as village councils. Of the 13 village councils in the sampling area, three union councils were randomly selected as the primary sampling unit. Subsequently, the researchers visited the primary health centers and designated vaccination centers within the chosen village councils. Newborn children were identified using the Extended Program for Immunization (EPI) register. Potential participants were appraoched with the help of Lady Health Workers (LHWs), community-based health workers linked to local health facilities. All families in the area were registered with a designated LHW who regularly visited them. Important information, such as the child’s name, father’s name, date of birth, and address, was recorded, and a list of eligible study participants was prepared. Once recruited, each participant will be followed up for two years. The study follows ethical principles involving human subjects as outlined in the declaration of Helsinki. Ethical approval for this study was granted by the Ethics Board of Khyber Medical University, Pakistan ref no DIR/KMU-EB/BR/001-03 dated 11/01/2024.

### Participants selection

The study population included 70 mother-infant pairs (dyads), according to the following criteria:

Inclusion criteria

• Apparently healthy infants of both genders aged 0 – 28 days
• Born by natural or caesarean delivery
• Born to parents from district Swat
• Parents/caregivers have no plans to move out of the stud site for at least two years after enrollment in the study.

The exclusion criteria are:

• Child born to an underage (<18 years old) mother.
• Infants born with severe acute or chronic medical conditions that require hospitalization, prolonged use of medication, or both, or diagnosed with enteropathies.
• Weight of the child is <1500 gm.

Ethical approval for the study was granted by the Research Ethics Board of Khyber Medical University. This study was also registered at www.clinicaltrial.gov (NCT05793294).

### Recruitment

In the local (Pashtun) culture, it is not possible to directly contact a potential female study participant without prior consent from the husband/male head of the household. Therefore, at each study site, an initial information session was held with the male parents/guardians with the help of elected local representatives and elders of the community. During the session, complete information about the study objectives, eligibility criteria, and data collection process was presented and explained by the team leader in the local (Pashto) language. A participant information sheet containing all the study details and procedures in an easy-to-understand, local (Pashto), and Urdu (national language of Pakistan) were also provided to the parents during the session. All queries were also answered. Following the session, eligible parents/guardians were invited by trained research assistants to enroll their babies in the study. Once they agreed, the parents were asked to sign a written informed consent form in their preferred language.

### Participants, funder and public involvement:

Community members, funder (NIH Pakistan) or the participants’ parents were not directly involved in developing the study design, conduct, and outcome measures. The study protocol was reviewed and approved by the Office of Research, Innovation, and Commercialization (ORIC) Khyber Medical University and National Institute of Health (NIH), Pakistan. Healthcare professionals (LHWs) involved in the study had close liaison with the community and acted as a bridge between the researchers and participants. They also contributed to the design of the data collection questionnaires, informed consent, and helped ensure confidentiality. However, the individual data were not reported to the participants.

### Study procedures

**Figure 1.**
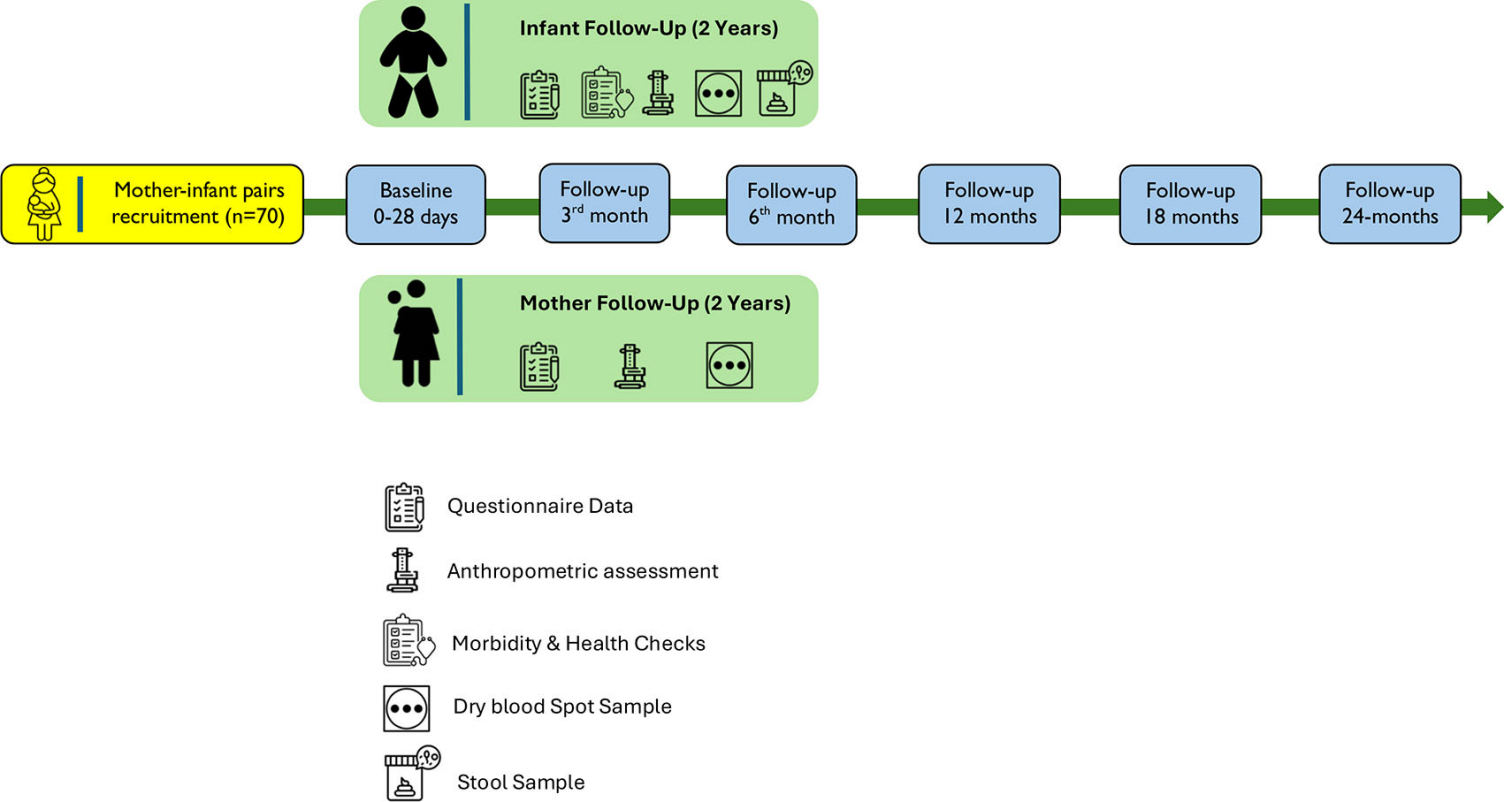
Flowchart of the study.

Figure 1 provides an overview of the overall study flow.

### Data collection

We will collect different data from both mother and infant at baseline and at each time point during the follow-up period. The data collection in this project used an Android version of the KoBoCollect/Toolbox as a data collection instrument. To collect the demographic and socioeconomic information of the study participants, an interviewer administered a structured questionnaire will be used. The questionnaire included information such as name, age, sex, household information, and socioeconomic status. Details about ante-natal and post-natal care will also be recorded using a structured, validated questionnaire adapted from the National Nutrition Survey Pakistan questionnaire. ^34^ These data were recorded only at baseline, that is, at the start of the study.

The Dietary Quality Questionnaire (DQQ) was used to assess the dietary intake of mothers. This method was chosen because, in Pakistan, there is no validated food frequency questionnaire to assess dietary intake. The DQQ is a standardized and easy-to-use tool to collect food group consumption data required for calculating diet quality indicators, such as Minimum Dietary Diversity for Women (MDD-W). The questionnaire has been validated and implemented across more than 100 countries including Pakistan. ^35^ The DQQ is a list-based method consisting of yes/no responses to 29 food groups consumed by the respondent during the previous day and night (24 hours). The procedure typically requires approximately 5–7 minutes to complete. Information regarding feeding practices of the children will be collected using validated, semi-structured questionnaires based on Infant and Young Child Feeding Practices (IYCF) indicators developed by the Technical Expert Advisory Group on Nutrition Monitoring (TEAM) of the WHO. ^36^ This set of simple, validated, and reliable indicators is widely used to assess IYCF practices across the globe, especially in low- and middle-income countries. The IYCF indicators were assessed by interviewing the mothers or the main caregivers. The mothers will also be interviewed to collect data about the child’s medical history, diarrhea and respiratory tract infection, hospitalization, and use of antibiotics using a structured questionnaire. DQQ, IYCF, and child health record data will be collected at baseline and at each time point during follow-up.

### Anthropometric assessment

At baseline and at each time point, the following anthropometric assessment will be performed on each child/infant and their mothers using standard methods. ^37^

**Height/length:** Recumbent length will be measured using an infantometer. Before measuring, the measuring board will be placed on a hard, flat surface such as a ground or sturdy table. The mother was asked to place the child gently on the board such that the infant’s head is aligned against the headboard while an assistant straightens the infant body and feet. When the child is position is correct, the researcher moves the foot board firmly against the child’s heels. The length of the child is recorded to the nearest 0.1 cm. The measurement will be repeated twice, and the average of the two measurements is recorded as the final length of the child. Mothers’ height will be recorded using a stadiometer following standard protocols. Briefly, they will be asked to remove shoes and gently stand on stadiometer. To ensure accurate measurement, the mothers will be instructed to stand up against the board, ensuring the Frankfurt plan position in which the back of the head, shoulder blades and buttocks and heels touch the back of the stadiometer. Once ready, the researcher will gently and firmly slide the measuring board’s moveable headpiece down until it touches the crown of the person’s head (compresses the hair). The procedure is repeated twice and the average of the two measurements is recorded as the height of the mother.

**Weight:** To record the weight of the child, a specialized electronic scale (Secca, UK) will be used. Before weighing the child, the parent will be instructed/helped to remove shoes, socks and heavy clothing except dry clean diapers or underpants from the child. When ready, the child is placed on his/her back on the center of the scale pan with the help of a trained researcher. We wait until the infant is still in the pan and the digital display is no more changing. The weight of the child is recorded to the nearest 0.01 kg. The weight of the mother is measured in kilograms using a calibrated electronic weighing scale. The mothers will be asked to remove shoes, jewellery and extra clothing (dupatta/chaddar) and stand calmly on the scale. The weight will be recorded to the nearest 0.01 kg twice and the average of the two measurement is considered the final weight of the mother.

**Mid upper arm circumference (MUAC):** For infant MUAC measurements, a non-stretchable, numbered and colored MUAC tape will be used. To prepare for the measurement, the infant mother will be asked to sit on a comfortable chair, place the child in her lap and remove any clothing covering the left hand of the child. The mid-point between the tip of the elbow and the shoulder is located by straightening the arm along the axis of the body and forearm at 90-degree forwards. Once the mid-point is marked with a pen, the arm is straightened and the MUAC tape is placed around the arm at the midpoint and the reading is taken to the nearest mm.

**Head circumference:** The head circumference of the child will be measured using a non-stretchable measuring tap around the most prominent part of head to the middle of the forehead while the hair and soft tissue is compressed. At least two measurements will be recorded and the average of the two will be taken.

### Sample collection

**Dry blood spot:** Dry blood spots (DBS) are collected from both the mother and the infant at baseline and during the follow-up by skin puncture of the third or fourth finger of the non-writing hand. ^38^ Before DBS collection, the participants’ hands were first warmed, followed by anterograde massaging of the finger in the direction of blood flow towards the puncture site. The DBS collection site, that is, the palmar side of the tip of the distal phalanx, was cleaned with 70% isopropyl alcohol and punctured using a single-use lacet. The first drop of blood was wiped off with a gauze pad, and subsequent drops were transferred to the marked circles on the surface of the filter paper without touching the surface. After drying, the DBS papers were transferred to a zip-lock bag and stored at -80 °C until further processing in the KMU main lab.

**Stool:** Stool samples are collected from the diapers of infants at each time point. For this purpose, the parents were provided diapers, a specimen collection jar, disposable gloves, a zip lock bag, and an instruction sheet one day before sample collection. The parents were instructed to regularly check their child’s diaper and remove it immediately after the child passed the stool. Stool samples were transferred immediately to the specimen container, placed in a zip-lock bag, and handed over to the research assistant within 4 h of sample collection. The research assistant transferred 400 mg of stool sample into 2 mL screw-top tubes prefilled with DNA shield reagent (Zymo Research USA) and sent it to the main KMU lab at room temperature, where it was stored at -80 °C.

### Laboratory analysis

**Hematological aassessment**

Dry blood samples will be used to assess the biomarkers of infections, micronutrient deficiencies, and inflammation. For this purpose, commercially available chemiluminscence-based Q-plex Aray kits (Quansys Biosciences) will be used following the manufacturer’s instructions. This assay reliably detects and quantifies histidine-rich protein II (biomarker of malarial infections), C-reactive protein, alpha-1-acid glycoprotein (biomarkers of inflammation), ferritin and soluble transferrin receptor (biomarker of iron deficiency), retinol binding protein (biomarker of vitamin A deficiency), and thyroglobulin (biomarker of iodine deficiency). ^39^

**DNA extraction and 16S rRNA sequencing**

Bacterial genomic DNA will be extracted from fecal samples (200 mg) stored at -80 °C using a ZymoBIOMICS DNA Miniprep Kit (Zymo Research, Irvine, CA, USA), following the manufacturer’s instructions. Each sample will be quantified using Denovix and quality was checked by PCR of the 16S rRNA gene target and agarose gel electrophoresis. For the amplicon PCR step, 12.5 ng of purified bacterial genomic DNA will be used to amplify the V3-V4 target region according to the manufacturer’s protocol. The amplified samples were then subjected to agarose gel electrophoresis to separate the DNA samples from the primer dimers. Each DNA band was extracted and purified using the QIAquick gel extraction kit (Cat. no. 28705; QIAGEN, Germany). After purification, the concentration of each amplicon PCR will be quantified using Nanodrop. Next, library preparation was performed via indexing PCR and the reaction was subsequently cleaned up and quantified, following the Illumina kit’s guidelines. The concentrations of all samples were then standardized to a minimum of 2.5 nmol. One microliter of DNA from each sample was pooled and sent for high-throughput sequencing on the Illumina Miseq ^®^ platform (Illumina, San Diego, California, USA) following the manufacturer’s instructions.

**Monitoring and evaluation strategy**

The monitoring and evaluation of the entire project will be conducted at all levels. Data entry will be randomly rechecked for accuracy by re-interviews or re-visits to households participating in the study. The principal investigator oversees the data collectors and enumerators. Bimonthly assessments will be performed by the project team consisting of the project manager, the PI, and the co-PI. In addition, the KMU ORIC team will monitor the overall progress of the project through its own monitoring and evaluation system. Any discrepancy or deviance from the main objectives or lapse in the timeframe and data collection procedures will be handled in close coordination of the project team with the field data collector and support from KMU.

### Participants retention strategies

A common issue in longitudinal cohort studies is systemic attrition, which can have a significant impact on the generalizability of the outcome of interest. Study participants may lose interest in the study and therefore decline their participation, especially in long-term cohort studies. Below is a list of the potential reasons for attrition in the current project and mitigation strategies.

1. The first and most important reason might be that some participants left the study district permanently and left the research team unable to track and collect data at different time points. To tackle this issue, we have set our inclusion criteria such that only those participants who are permanent residents of these districts and who confirm that they have no plans to move outside the district permanently will be recruited.
2. Reducing barriers to participation by hiring local research assistants and their training, ensuring anonymity, translating the data collection tools to the local language (Pashto), and pilot testing.
3. A follow-up and reminder strategy was set up by calling the participants before data collection.
4. Tracing via alternative phone numbers and visiting the house physically when participants did not respond.

### Data analysis

All *in sillico* data processing and analysis will be performed on the IBU Linux Cluster for Rocky Linux 8 from the servers of the University of Bern on a local machine running Ubuntu 20.04.06 LTS (32GB, Intel ^®^ Core™ i7-9750H CPU @ 2.60GHz × 12) and within R (4.4.1 2024-06-14) package environments in RStudio (2024.04.2). Sequence reads obtained for each sample were processed for quality using FastQC (v0.11.9) and MultiQC (version 1.11). Further quality checks, read trimming, chimera removal, denoising, and taxonomy assignment (using a trained Naive Bayes kmer classifier) will be performed directly from within the QIIME2 workflow (q2cli version 2024.5.0) using the included DADA2 software. Standard core metrics such as alpha diversity indices (Faith’s richness, Simpson’s evenness, and Shannon index) and beta diversity (Bray-Curtis, weighted UniFrac for the quantitative metrics; jaccard and unweighted UniFrac for the qualitative ones; Aitchison) will be computed with QIIME2, then explored in Qiime2View, and further processed and compared with Phyloseq and qiime2R packages in R. We will use the Emperor biplot plugin, Principal Coordinate Analysis (PCoA), and beta-group-significance plugin to test beta diversity significance and characterize the top bacterial genera that explain the microbiota distributions across the samples.

We will seek to identify any correlations between the collected metadata and microbiome composition, and identify any gut/oral microbiota trends associated with nutritional status and food diversity. We decorticate the variations in the observed microbiota composition across different groups, such as weight, height, age, and nutritional status. For alpha diversity comparisons, the groups’ significance was computed and tested using Kruskal-Wallis pairwise tests. We will also use the q2-longitudinal plugin to investigate longitudinal and paired sample comparisons, that is, to determine if or how samples change between the different time points and treatments/nutrients. All postprocessing and statistical analyses will be performed in R.

Weight for height, weight for age, and height for age z-score will be calculated using the WHO Anthroplus software and compared with microbiome composition using R.

### Dissemination

The results of the study will be disseminated to the public and academia, including our research team and collaborators, scientists, and the broader research community via participation in scientific conferences, meetings, and publications in peer-reviewed journals.

### Study status

The study began in 2024 and will continue until May 2026. As of October 2024, 70/70 (100%) were recruited. Data and samples were collected for the baseline (n=70) and first follow-up periods (3 months; n = 66).

## Discussion

The CHAMP study aimed to longitudinally characterize gut microbiome development in newborn infants during the first two years of life and included 70 mother-infant pairs (dyads) recruited from remote, rural communities of District Swat, Pakistan. This study involved comprehensive data collection regarding dietary intake, nutritional status, infant growth, and morbidities at different time points. Prospective collection of stool samples will allow gut microbiome assessment over time and identify demographic, maternal, and child-related factors affecting its diversity and abundance. The study findings will contribute towards a better understanding of gut microbiome colonization and development, associated factors, and its impact on growth in infants from malnourished areas of Pakistan.

Microbial colonization of the gut during infancy plays a crucial role in establishing the intestinal barrier and developing the immune system. ^40^ Numerous studies have reported rapid changes in the composition and diversity of the microbiota during the first two years of life in healthy infants. However, the pattern and extent of microbial colonization are largely influenced by feeding practices and nutritional status of infants. A bidirectional relationship exists between the development of the gut microbiome and nutritional status. Malnutrition can affect gut microbiome development and maturation during early childhood, resulting in dysbiosis. As a result, an immature and altered gut microbiome leads to malnutrition owing to impaired energy production, vitamin biosynthesis, and immune dysfunction. ^41, 42^ These findings are critical, especially in developing countries such as Pakistan, where childhood malnutrition is a public health issue. ^34^ The CHAMP study will help to dissect the relationships between dietary intake, nutritional status, and gut microbiome phenotypes during infancy and pave the way for developing microbiome-based interventions to tackle the problem of malnutrition in Pakistan.

Our study is the first to characterize gut microbiome development in newborn infants from malnourished areas of Pakistan. The study will greatly help to understand infant growth trajectory in relation to gut microbiome development and provide a conceptual basis for sustainable nutrition recommendations and interventions in the future. The prospective longitudinal nature of the study will help in the collection of detailed data with minimal constraints (six visits in 2 years) and associated risks (no intervention and minimally invasive procedures to collect biological samples). This study will obtain precise data on dietary intake and nutritional status and their short-term and medium-term impacts on gut microbiome development, and vice versa. To gain deeper insights into how these factors, especially dietary intake patterns and socioeconomic status, impact gut microbiome development, we will compare our study results with similar studies conducted in Zimbabwe, a low-income country (The University of Zimbabwe College of Health Sciences (UZ-CHS) Birht Cohort) ^43^ and Switzerland, a high-income country (The Bern Birth Cohort – BeBiCo study). ^44^ Both of these studies involved mother-infant pairs and collected neonatal data on demographics, socioeconomic status, and diet at time points corresponding to our study. These studies also collected fecal samples, which will allow us to compare gut microbiome development in Pakistani infants with infants from Switzerland and Zimbabwe, countries with completely different landscapes in terms of environmental, cultural, and dietary intake patterns.

Our study has some limitations. First, the launch and implementation of a cohort study in remote, difficult-to-access rural areas are challenging, anywhere in the world, including Pakistan. As a result, dropout and attrition in the sample size are expected. Second, the study involved multiple assessments, requiring the mother to travel long distances in mountainous areas to reach the sampling site. The process is time-consuming and potentially burdensome to the mother and infants. However, these issues will be partly addressed by providing compensation for travel and sending advance reminders about follow-up visits using phone calls. Third, due to the self-reported and retrospective nature of the data collection during each visit, response and social desirability bias cannot be fully avoided. Finally, we could only identify the association between growth and gut microbiome development, but no causal relationship because of the non-interventional study design.

### Ethical considerations

The study follows ethical principles involving human subjects as outlined in the declaration of Helsinki. Ethical approval for this study was granted by the Ethics Board of Khyber Medical University, Pakistan ref no DIR/KMU-EB/BR/001-03 dated 11/01/2024.

### Consent statement

In the local (Pashtun) culture, it is not possible to directly contact a potential female study participant without prior consent from the husband/male head of the household. Therefore, at each study site, an initial information session was held with the male parents/guardians with the help of elected local representatives and elders of the community. During the session, complete information about the study objectives, eligibility criteria, and data collection process was presented and explained by the team leader in the local (Pashto) language. A participant information sheet containing all the study details and procedures in an easy-to-understand, local (Pashto), and Urdu (national language of Pakistan) were also provided to the parents during the session. All queries were also answered. Following the session, eligible parents/guardians were invited by trained research assistants to enrol their babies in the study. Once they agreed, the parents were asked to sign a written informed consent form in their preferred language.

## Data Availability

No data are associated with this article. Muhammad, Shahzad, 2024, “Child health, nutrition and gut microbiota development during the first two years of life; study protocol of a prospective cohort study from the Khyber Pakhtunkhwa, Pakistan”, https://doi.org/10.7910/DVN/ROU3VJ ^45^ This project contains the following extended data:
• Data Collection Quarionnaire.doc Data are available under the terms of the Creative Commons Zero “No rights reserved” data waiver (CC0 1.0 Public domain dedication).
