# Supplementary material for: Child health, nutrition and gut microbiota development during the first two years of life; study protocol of a prospective cohort study from the Khyber Pakhtunkhwa, Pakistan
Source: F1000Res. 2025 Aug 8;13:1336. Originally published 2024 Nov 7. [Version 2] doi: 10.12688/f1000research.158415.2 (PMC12449683; doi:10.12688/f1000research.158415.2)
Supplement: Peer review history

# Reviewer report: Reviewer response for version 2

**DOI:** 10.5256/f1000research.186009.r407052

**Published:** 2025-09-19

**Authors:** Roshonda B Jones

**Affiliations:** 1 North Carolina Agricultural and Technical State University, Greensboro, North Carolina, USA

The authors of "Child health, nutrition and gut microbiota development during the first two years of life; study protocol of a prospective cohort study from the Khyber Pakhtunkhwa, Pakistan" provide a compelling argument for the need to study the developing gut microbiome in children in Pakistan, given the high rates stunting/wasting/micronutrient deficiencies in this population, which are all directly related to the gut microbiome. To examine this, it is necessary to characterize the gut microbiome in this population to understand how to use nutritional interventions to address malnutrition. Here, they present the study protocol that they will use to characterize the gut microbiome in this population.

 Here a few minor comments:

 1) This second paragraph of the section "Study design and setting" is repeated in the section "Sampling technique". Maybe remove completely or mention only briefly. As it stands currently,  it feels like the reader has to read the same information back to back.

 2) Figure 1: As it stands, the figure makes it seem that the follow up measures will only be taken at the 2 year visit.  I assume it is also happening at the 3M, 6M, 12M also 18M. I would make this clearer in the Figure. Maybe “Infant Follow-Up (2 years)” can be “Infant Follow up 3M, 6M, 12M, 18M and 24M” and the box spans the entire arrow. Also add in something that states what is being collected at baseline.

 3) In the section " **Monitoring and evaluation strategy",  **it reads “Data entry will be randomly rechecked for accuracy by re-interviews or re-visits to households participating in the study”. How long after a visit will these random rechecks happen? How will the investigators make sure that differences in answers are due to the responses being taken later in time or that there was a collection error the first time responses were recorded.

 4) In the Data Analysis section, when an R package is mentioned make sure that you state "R package" instead of just "package" so as to not assume that the reader knows it is an R package.

Is the study design appropriate for the research question?

Yes

Is the rationale for, and objectives of, the study clearly described?

Yes

Are sufficient details of the methods provided to allow replication by others?

Yes

Are the datasets clearly presented in a useable and accessible format?

Not applicable

Reviewer Expertise:

I am a microbial computational biologist focusing on the developing microbiome in children.

I confirm that I have read this submission and believe that I have an appropriate level of expertise to confirm that it is of an acceptable scientific standard.

# Reviewer report: Reviewer response for version 2

**DOI:** 10.5256/f1000research.186009.r403885

**Published:** 2025-09-12

**Authors:** Ryan Marsh

**Affiliations:** 1 Department of Applied Sciences, Northumbria University, Newcastle,, UK

I am pleased to find that the authors have addressed my previous comments. This includes clarity surrounding the study design and data collection, alongside comprehensive detail upon the *now *altered sequencing approach that further accommodates predicted functionality of microbial communities sequenced.

 As for other suggestions regarding sampling, I understand the cultural implications as discussed by the authors with respect to various anatomical sites, but applaud the decisions to " *collect oral swab samples from both mothers and infants. These samples are now systematically collected at each scheduled time point."*

 Overall, I am pleased with the changes and eagerly await results from the study - best of luck!

Is the study design appropriate for the research question?

Partly

Is the rationale for, and objectives of, the study clearly described?

Yes

Are sufficient details of the methods provided to allow replication by others?

Partly

Are the datasets clearly presented in a useable and accessible format?

Not applicable

Reviewer Expertise:

Gut microbiome research (Cystic Fibrosis), utilising multi-omics.

I confirm that I have read this submission and believe that I have an appropriate level of expertise to confirm that it is of an acceptable scientific standard.

# Reviewer report: Reviewer response for version 1

**DOI:** 10.5256/f1000research.173997.r358559

**Published:** 2025-02-05

**Authors:** Ryan Marsh

**Affiliations:** 1 Department of Applied Sciences, Northumbria University, Newcastle,, UK

Dear Authors,

 Many thanks for the opportunity to review this work. Please find below my comments to each section of the report.

**Is the rationale for, and objectives of, the study clearly described? (YES)**

 The Study Protocol presented here by Shahzad and colleagues undoubtedly addresses the warranted need to further understand early life microbial dynamics across infants from malnourished areas of Pakistan. Elucidating the relationships between the microbiome and early lifestyle factors will no doubt increase favourable outcomes throughout development in children from such regions.

 I have no qualms with the study rationale, and the body of literature cited for this work is of high quality and relatable to the study background.

**Is the study design appropriate for the research question? (PARTLY)**

 Overall, I am happy with the study design, which provides a logical narrative. I understand that sampling may have already begun, but I would double check tense usage throughout just to be consistent and to amend any potential language errors.

 I understand a variety of questionnaires will be/are being used by the researchers to collect the relevant demographic information. Some clarity would be appreciated on the following:

Study Procedures, Data collection: First paragraph reads at the end: “These data were recorded only at baseline, that is, at the start of the study”. Does this refer only to the original demographic/socio-economic data, and *not* the ante-natal and post-natal care information? If the latter *is* included, I would highly recommend the authors specify examples of what this may encompass, as I believe elements of these may overlap with other questionnaires poised by the authors, such as antibiotic usage.

 As for other areas of the sampling:

In-person sampling: It would be good to set or specify if there is a time-standardisation here upon arrival, as the limitations describe the burdensome journey that will have to be made by participants. This would be applicable to dry blood spot testing if this is to commence following this journey, which may skew data on inflammatory outcomes etc.

Sample collection: Given the authors highlight the potential translocation of bacterial taxa from various anatomical sites between mother and newborn, which specifies the gut of the (line 10, Introduction), it would have been very interesting to investigate the microbiota of the mother as close to the infants’ baseline sample as possible, to decipher if common seeding patterns that associate with favourable growth outcomes etc. Not sure if this can be feasibly integrated given the original ethical approvals put in place, but food for thought no doubt.

**Are sufficient details of the methods provided to allow replication by others? (PARTLY)**

 Overall, I am happy with the methods provided.

 For the proposed approach for investigation into microbial communities utilising 16S rRNA amplicon sequencing on the Illumina Miseq® platform, I would ask for the following information, as to more clearly allow methods to be replicated by others:

DNA extraction and 16S rRNA sequencing: “Next, library preparation was performed via indexing PCR and the reaction was subsequently cleaned up and quantified, following the Illumina kit’s guidelines”. Can you please elaborate on which kit in particular is used for the second-step PCR and subsequent library clean-up, or was this done manually whilst referring to Illumina’s “16S Metagenomic Sequencing Library Preparation” document? If possible, I would also highly recommend the researchers quantify DNA using fluorometric approaches, such as the Qubit fluorometer if manually adjusting and normalising as hinted in the protocol, to ensure adequate sampling read depth across all participants.

DNA extraction and 16S rRNA sequencing: Given that V3-V4 region is the target of interest, I am assuming that this will be performed using 2 × 300 bp, V3 chemistry? I would always recommend highlighting this if possible. From personal experience I have found that the reverse read (R2) can significantly drop-off on these kits. As the background literature cites differences at the genus level across the microbiome across infants from both developed, and developing studies, I might suggest  V2 Chemistry targeting the V4 region (2 × 250 bp) to increase robustness of read depth and quality across samples, if genus-level comparisons are sufficient for the authors.

DNA extraction and 16S rRNA sequencing: Mention of sequencing controls (and any positive mock community controls) would be useful.

 As for the wider interpretations and considerations of the results/data:

Data analysis: The authors describe the relationships they are looking to explore between microbiota diversity and community composition with participant demographics they are looking to collect. Within the “data collection” section the protocol, it states: “The mothers will also be interviewed to collect data about the child’s medical history, diarrhea and respiratory tract infection, hospitalization, and use of antibiotics using a structured questionnaire”. I would anticipate the researchers to also investigate relationships between antibiotic usage and microbiota structure, but there is no specific mention of this (unlike nutritional status, food diversity, height, weight, age, etc..). Perturbation of intestinal microbial communities following antibiotic administration is well documented, which is of heightened importance during the developmentally critical age window (0-2 years) of the infants in this study. Additionally, I understand from the literature, that over-the-counter dispensation of antibiotics can be common in Pakistan which may increase the likelihood of a significant perturbation events to the intestinal microbiota.

**General formatting (PARTLY)**

 Type errors:

 “ouncils” should read “councils” – Sampling technique paragraph, line 4.

 “measuring tap” should read “measuring tape” – Anthropometric assessment paragraph, line 1.

 “Hematological aassessment” – error in title of this section in the Laboratory analysis.

 Overall, I would advocate for the acceptance of the study protocol once these minor changes are implemented. I look forward to reading the final indexed  study. Great work so far, and good luck moving forward!

 Best,

 Dr. Ryan Marsh

Is the study design appropriate for the research question?

Partly

Is the rationale for, and objectives of, the study clearly described?

Yes

Are sufficient details of the methods provided to allow replication by others?

Partly

Are the datasets clearly presented in a useable and accessible format?

Not applicable

Reviewer Expertise:

Gut microbiome research (Cystic Fibrosis), utilising multi-omics.

I confirm that I have read this submission and believe that I have an appropriate level of expertise to confirm that it is of an acceptable scientific standard, however I have significant reservations, as outlined above.

## References

[1] : Antibiotic dispensation without prescription by community pharmacies in Pakistan. *Journal of Medicine, Surgery, and Public Health* .2024;2: 10.1016/j.glmedi.2024.100065 10.1016/j.glmedi.2024.100065

# Response

**Published:** 2025-08-01

**Authors:** Shahzad Muhamamd

**Affiliations:** Khyber Medical University, Pakistan

**Author Response**

 Aug 1st, 2025

 Muhammad Shahzad

 Associate Professor, Institute of Basic Medical Sciences, Khyber Medical University, Peshawar, Pakistan

 F1000 Peer Review Journal

 Ref: Response to Peer Reviewers’ Reports - F1000 Research Peer Review “Child health, nutrition and gut microbiota development during the first two years of life; study protocol of a prospective cohort study from the Khyber Pakhtunkhwa, Pakistan”.

 Dear F1000 Research Editorial Team,

 Thank you for facilitating the peer review process for our manuscript. We have carefully addressed the reviewers’ comments, and we believe that the revised manuscript now aligns with the journal’s requirements. Below, we provide a line-by-line response to the reviewers’ comments, with the original comments highlighted in bold.

**Is the rationale for, and objectives of, the study clearly described? (YES)**

**The Study Protocol presented here by Shahzad and colleagues undoubtedly addresses the warranted need to further understand early life microbial dynamics across infants from malnourished areas of Pakistan. Elucidating the relationships between the microbiome and early lifestyle factors will no doubt increase favourable outcomes throughout development in children from such regions.**

**I have no qualms with the study rationale, and the body of literature cited for this work is of high quality and relatable to the study background.**

*Our response: The authors thank the reviewer for taking time out to review our manuscript and enhance the quality of our work.*

**Is the study design appropriate for the research question? (PARTLY)**

**Overall, I am happy with the study design, which provides a logical narrative. I understand that sampling may have already begun, but I would double check tense usage throughout just to be consistent and to amend any potential language errors.**

*Our response: Thanks for your comments. In the revised manuscript, tense usage is double checked and corrected accordingly.*

**I understand a variety of questionnaires will be/are being used by the researchers to collect the relevant demographic information. Some clarity would be appreciated on the following:**

**Study Procedures, Data collection: First paragraph reads at the end: “These data were recorded only at baseline, that is, at the start of the study”. Does this refer only to the original demographic/socio-economic data, and not the ante-natal and post-natal care information? If the latter is included, I would highly recommend the authors specify examples of what this may encompass, as I believe elements of these may overlap with other questionnaires poised by the authors, such as antibiotic usage.**

*Our response: Thanks for the suggestion. It refers to both sociodemographic data and ante-natal and post-natal care data as clarified in the revised manuscript.*

**As for other areas of the sampling:**

**In-person sampling: It would be good to set or specify if there is a time-standardisation here upon arrival, as the limitations describe the burdensome journey that will have to be made by participants. This would be applicable to dry blood spot testing if this is to commence following this journey, which may skew data on inflammatory outcomes etc.**

*Our response. Thank you for your valuable feedback. Upon arrival at the study site at each scheduled time point, participants first undergo formal identification procedures. Subsequently, stool samples collected at home are processed, followed by comprehensive data collection and anthropometric assessments. These procedures require a minimum of one hour to complete. Thus, participants are provided with adequate time to rest prior to the collection of oral swab and dried blood spot (DBS) samples.*

**Sample collection: Given the authors highlight the potential translocation of bacterial taxa from various anatomical sites between mother and newborn, which specifies the gut of the (line 10, Introduction), it would have been very interesting to investigate the microbiota of the mother as close to the infants’ baseline sample as possible, to decipher if common seeding patterns that associate with favourable growth outcomes etc. Not sure if this can be feasibly integrated given the original ethical approvals put in place, but food for thought no doubt.**

*Our response: Thank you for this valuable suggestion. While we fully acknowledge the importance of maternal microbiota, cultural barriers have unfortunately limited our ability to collect microbiota samples from mothers. However, recognizing its significance, we have obtained ethical approval to collect oral swab samples from both mothers and infants. These samples are now systematically collected at each scheduled time point.*

**Are sufficient details of the methods provided to allow replication by others? (PARTLY)**

**Overall, I am happy with the methods provided.**

**For the proposed approach for investigation into microbial communities utilising 16S rRNA amplicon sequencing on the Illumina Miseq® platform, I would ask for the following information, as to more clearly allow methods to be replicated by others:**

**DNA extraction and 16S rRNA sequencing: “Next, library preparation was performed via indexing PCR and the reaction was subsequently cleaned up and quantified, following the Illumina kit’s guidelines”. Can you please elaborate on which kit in particular is used for the second-step PCR and subsequent library clean-up, or was this done manually whilst referring to Illumina’s “16S Metagenomic Sequencing Library Preparation” document? If possible, I would also highly recommend the researchers quantify DNA using fluorometric approaches, such as the Qubit fluorometer if manually adjusting and normalising as hinted in the protocol, to ensure adequate sampling read depth across all participants.**

**DNA extraction and 16S rRNA sequencing: Given that V3-V4 region is the target of interest, I am assuming that this will be performed using 2 × 300 bp, V3 chemistry? I would always recommend highlighting this if possible. From personal experience I have found that the reverse read (R2) can significantly drop-off on these kits. As the background literature cites differences at the genus level across the microbiome across infants from both developed, and developing studies, I might suggest  V2 Chemistry targeting the V4 region (2 × 250 bp) to increase robustness of read depth and quality across samples, if genus-level comparisons are sufficient for the authors.**

**DNA extraction and 16S rRNA sequencing: Mention of sequencing controls (and any positive mock community controls) would be useful.**

*Our response: Thanks for the detailed feedback and suggestions. We have decided to perform shotgun metagenomic sequencing as it is more reliable than 16s sequencing. Please see revised manuscript for detail changes in the methodology and data analysis.*

**As for the wider interpretations and considerations of the results/data:**

**Data analysis: The authors describe the relationships they are looking to explore between microbiota diversity and community composition with participant demographics they are looking to collect. Within the “data collection” section the protocol, it states: “The mothers will also be interviewed to collect data about the child’s medical history, diarrhea and respiratory tract infection, hospitalization, and use of antibiotics using a structured questionnaire”. I would anticipate the researchers to also investigate relationships between antibiotic usage and microbiota structure, but there is no specific mention of this (unlike nutritional status, food diversity, height, weight, age, etc..). Perturbation of intestinal microbial communities following antibiotic administration is well documented, which is of heightened importance during the developmentally critical age window (0-2 years) of the infants in this study. Additionally, I understand from the literature, that over-the-counter dispensation of antibiotics can be common in Pakistan which may increase the likelihood of a significant perturbation events to the intestinal microbiota.**

*Our response: Infants Gut microbiome diversity and composition will be assessed in relation to the demographic characteristics, dietary intake and nutritional status of both the mother and infant and infants health record including diarrheal episodes, RTI and antibiotic use history and duration. Please see revised manuscript for details.*

 General formatting (PARTLY)

 Type errors:

 “ouncils” should read “councils” – Sampling technique paragraph, line 4.

 Corrected

 “measuring tap” should read “measuring tape” – Anthropometric assessment paragraph, line 1.

 “Hematological aassessment” – error in title of this section in the Laboratory analysis.

 Corrected
